# Resource Deployment in Response to Trauma Patients

**DOI:** 10.7759/cureus.49979

**Published:** 2023-12-05

**Authors:** Thomas Matthews, Alexa LaScala, Theresa Tomkin, Lisa Gaeta, Karen Fitzgerald, Michele Solomita, Barbara Ragione, Taslima P Jahan, Saliha Pepic, Lillian Apurillo, Victoria Siegel, Amy Frederick, Abenamar Arrillaga, Lauren R Klein, John Cuellar, Christopher Raio, Keri Penta, Lauren Rothburd, Sarah A Eckardt, Patricia Eckardt

**Affiliations:** 1 Nursing, Good Samaritan University Hospital, West Islip, USA; 2 Quality Improvement, Good Samaritan University Hospital, West Islip, USA; 3 Nursing Administration, Good Samaritan University Hospital, West Islip, USA; 4 Research, City University of New York, New York, USA; 5 Research, Molloy University, Rockville Center, USA; 6 Nursing Education, Molloy University, Rockville Center, USA; 7 Trauma, Good Samaritan University Hospital, West Islip, USA; 8 Surgical Critical Care, Good Samaritan University Hospital, West Islip, USA; 9 Emergency Medicine, Good Samaritan University Hospital, West Islip, USA; 10 Orthopedic Surgery, Good Samaritan University Hospital, West Islip, USA; 11 Nursing/Performance Improvement, Good Samaritan University Hospital, West Islip, USA; 12 Data Scientist, Eckardt & Eckardt Consulting, LLC, St. James, USA

**Keywords:** geriatric trauma, trauma team, falls trauma, trauma deployment, trauma activation, emergency staff deployment

## Abstract

Background

Variance in the deployment of the trauma team to the emergency department (ED) can result in patient treatment delays and excess burden on ED personnel. Characteristics of trauma patients, including mechanism of injury, injury type, and age, have been associated with differences in trauma resource deployment. Therefore, this retrospective, single-site study aimed to examine the deployment patterns of trauma resources, the characteristics of the trauma patients associated with levels of trauma resource deployment, and the deployment impact on ED workforce utilization and non-trauma ED patients.

Methodology

This was an investigator-initiated, single-institution, retrospective cohort study of all patients designated as a trauma response and admitted to a community hospital’s ED from July 01, 2019, through July 01, 2022.

Results

Resource deployment for trauma patients varied by mechanism of injury (p < 0.001), injury type (p < 0.001), and patient age groups (p < 0.001). Specifically, there was a lower average trauma activation for geriatric trauma patients with a fall as a mechanism of injury compared to all younger patient groups with any mechanism of injury (F(5) = 234.49, p < 0.001). In the subsample, there was an average of 3.35 ED registered nurses (RNs) allocated to each trauma patient. Additionally, the ED RNs were temporarily reallocated from an average of 4.09 non-trauma patients to respond to trauma patients, despite over a third of the trauma patients in the subsample being the trauma patients being discharged home from the ED.

Conclusions

Trauma activation responses need to be standardized with a specific plan for geriatric fall patients to ensure efficient use of trauma and ED personnel resources.

## Introduction

Recent studies have examined the impact of inappropriate trauma team response on patient outcomes, trauma team members’ utilization, and trauma center resources [1-7]. Underdeployment of the trauma team in the emergency department (ED) can result in delays in patient triage and treatment, increased risk for complications, and increased mortality [3,8,9]; while overdeployment can also increase patient risk, staff burnout, and excess burden on trauma center resources [2,4,10]. Additionally, disparities in the deployment of resources have been noted within certain patient populations triaged to lower levels of trauma response (e.g., geriatric, blunt non-penetrating trauma victims) [11-14].

To date, there is a dearth in the literature examining disparities in deployment among certain populations triaged to a lower level of trauma response [12,13,15,16] and the frequency and impact of additional ED workforce, such as ED staff registered nurses (RNs), deployed for trauma patient care [17-19]. Overall, research protocols use the metrics established in the American College of Surgeons Trauma Quality Programs (TQP) Best Practices guidelines, such as trauma team activation, as proxy measures of the appropriateness of staff deployment for trauma patients [20,21]. However, particularly in non-tertiary care settings, additional ED personnel are often deployed to triage and treat trauma patients in addition to the activated trauma team members [7,9,22,23]. The regular use of these resources can have a significant impact on maintaining quality patient outcomes and staff satisfaction. Moreover, data on the use of non-trauma team ED personnel to care for trauma patients can provide evidence for future appropriate ED staffing models [4,24,25].

Objective(s)/specific aims and hypotheses

Therefore, this study aimed to (1) examine the deployment of trauma resources and the characteristics of the trauma patients where these resources are applied; (2) describe the frequency and characteristics of ED nurse deployment to augment trauma team activation and the characteristics of the trauma patients where these resources are applied; (3) explore the impact of ED nurse deployment to trauma patients on ED resources.

## Materials and methods

This was an investigator-initiated, single-institution, retrospective cohort study of all patients designated as a trauma response and admitted to a community hospital’s ED from July 01, 2019, through July 01, 2022. The additional data from an existing quality improvement (QI) project measuring ED nurse time motion study included a subpopulation of the total sample. The subpopulation data were aggregated by ED nurses’ assignment area for the date and measured minutes in deployment and number of ED nurses deployed from non-trauma assignment to a specific trauma patient during a two-week period in March and April 2021.

After obtaining approval from the institutional review board on September 30, 2022, for research with human subjects, the data were retrospectively abstracted from the National Trauma Data Registry and the patients’ electronic medical records (EMRs). All patients who were designated as a trauma response and admitted to a level 2 trauma center’s ED from July 01, 2019, through July 01, 2022, were included in the sampling frame.

Statistical analysis

The analysis was completed using SPSS Statistics for Windows, version 28 (IBM Corp., Armonk, NY, USA) and Stata Statistical Software: Release 18 (StataCorp LLC, College Station, TX, USA). The Reporting of studies Conducted using the Observational Routinely collected health Data (RECORD) guidelines were used for reporting study results. Independent sample t-tests and one-way analyses of variance were used to compare the means between groups (trauma activation levels and patient demographics and clinical variables) on the continuous variables. Chi-square (χ^2^) tests were used to compare proportional differences between the groups (trauma activation levels and patient demographics and clinical variables) on the nominal variables. Fisher’s exact test was used to analyze categorical data if more than 20% of cells had expected counts of less than 5. Inferential analyses used a two-tailed approach, and 0.05 was chosen as the a priori critical alpha. Data were retrieved from the National Trauma Data Registry and the patients’ EMR. All patients designated as a trauma response and admitted to a level 2 trauma center’s ED from July 01, 2019, through July 01, 2022, were included in the sample. Patient data included age, diagnoses at admission and during their stay, ED arrival time, length of stay in the ED, mechanism of injury (MOI), trauma activation level, and ED disposition. Additionally, the study categorized diagnoses using the International Classification of Diseases groups, as well as the most common categories for MOIs such as falls, motor vehicle accidents/collisions (MVAs-MVCs), and all others.

Activation levels were defined using the four levels of the institution’s trauma activation protocol (levels 1-4), with level 1 being the highest level and 4 referring to no trauma alert. Data with a trauma activation level of “N” = level 4 and were defined as not activated. Trauma activation levels of “C” = level 3 and indicated a “Consult,” which, as defined by this hospital, required a response within one hour by an advanced practice provider or surgeon. Trauma activation levels of “2” were classified as “Trauma Alert,” which required a surgeon to respond within 30 minutes, as defined by this hospital. The highest trauma activation “1” is a “Code T” and required a surgeon to respond within 15 minutes of activation minutes, which is an American College of Surgeons requirement. Patients were stratified into four groups based on their activation levels. Data were then compared to determine if there were any differences among significant variables such as age, ED length of stay, ED arrival time, MOI, trauma activation time, trauma-related diagnoses, and ED disposition. Additionally, to explore possible disparities in the deployment of resources associated with lower levels of triage and trauma response activation for geriatric, blunt non-penetrating trauma victims, patients were grouped by age (0-14,15-24,25-44, 45-64,65-84, ≥85 years of age) and MOI.

## Results

The sample (n = 6,404) comprised 5,638 (88.04%) adults (≥18 years of age), with a majority of patients being white (n = 4,154, 64.90%) and non-Hispanic/Latino (n = 5,141, 80.50%). The predominant MOI was falls (n = 3,417, 53.36%), followed by MVAs (n = 983, 15.35%). These findings were also compared to categorical data for trauma type; the majority of patients were primarily classified as blunt trauma (n = 6,134, 95.80%). Additionally, the majority of patients were admitted to the ED during the night shift (n = 3,044, 47.53%), arrived from home (n = 3,389, 52.90%), and arrived at the ED via ground ambulance (n = 4,564, 71.28%). The activation level for most patients was a “consult,” defined by the hospital as needing a response within one hour by an advanced practice provider or surgeon. Patients in the sample were admitted to the floor the majority of the time (n = 3,321, 51.86%). On average, pediatric patients spent less time in the ED (4.98 hours, SD = ±2.27) compared to adult patients (7.99 hours, SD = ±4.94) (Table 1).

**Table 1:** Sample characteristics for trauma patients admitted to the ED (n = 6,404). **: Data formatted as n, percentage (n, %) for categorical data, and mean, standard deviation (x̄, ±). ED: emergency department; n: sample size; SD: standard deviation; MD: medical doctor; MVA: motor vehicle accident; NEC: not elsewhere classifiable; APP: advanced practice provider; ACS: American College of Surgeons; AMA: against medical advice

Variable	Statistic**
Age	50.84 (SD = 27.81)
Pediatric trauma, n = 766 (11.96%)	7.03 (SD = 4.8)
Adult trauma, n = 5,638 (88.04%)	56.79 (SD = 24.07)
Race	
American Indian/Alaska Native	2 (<1.00%)
Asian	67 (1.00%)
Black	794 (12.40%)
Native Hawaiian/Other Pacific Islander	3 (00.00%)
Other	1380 (21.60%)
White	4,154 (64.90%)
Ethnicity	
Non-Hispanic/Latino	5,141 (80.50%)
Hispanic/Latino	1,245 (19.50%)
Trauma type	
Blunt	6,134 (95.80%)
Burns	77 (1.20%)
Penetrating	192 (03.00%)
Arrived from	
Clinical/MD Office	42 (00.70%)
Home	3,389 (52.90%)
Jail	1 (00.00%)
Nursing home	159 (02.50%)
Referring hospital	291 (04.50%)
Scene	2180 (34.10%)
Super living	109 (1.70%)
Urgent care	231 (03.60%)
Transport arrival type	
Ground ambulance	4,564 (71.28%)
Police	12 (00.19%)
Private/Public vehicle/Walk-in	1,827 (28.53%)
ED arrival time/Shift	
Day (0700–1459)	2,311 (36.09%)
Night (1500–2259)	3,044 (47.53%)
Overnight (2300–0659)	1,049 (16.38%)
Mechanism of injury – category	
Cut/Pierce	327 (5.11%)
Drowning	18 (0.28%)
Fall	3,417 (53.36%)
Burn	74 (1.16%)
Firearm	83 (1.3%)
Machinery	54 (0.84%)
Medical care	1 (0.02%)
MVA-Other	983 (15.35%)
MVA-Motorcycle	168 (2.62%)
MVA-Pedestrian	304 (4.75%)
Natural/Environmental	111 (1.73%)
Other specified, class	170 (2.65%)
Other specified, NEC	29 (0.45%)
Other transport	44 (0.69%)
Overexertion	11 (0.17%)
Pedacyclist-Other	108 (1.69%)
Pedestrian-Other	16 (0.25%)
Poison	2 (0.03%)
Struck by or against	458 (7.15%)
Trauma activation time	
Trauma activation level before arrival	817 (12.76%)
Trauma activation level after arrival	4,110 (64.18%)
Activation level	
N = Not activated; trauma not notified by the patient; trauma aware but not activated; usually minor/isolated injuries	1,467 (22.91%)
C = Consult – requires a response within one hour by APP or surgeon – defined by the hospital	2,481 (38.74%)
2 = Trauma alert – requires the surgeon to respond within 30 minutes – defined by the hospital	1,894 (29.58%)
1 = Code T – the highest level of activation – requires the surgeon to respond within 15 minutes – ACS requirement	557 (8.7%)
ED length of stay (hours)	7.63 (SD = 4.8)
Pediatric trauma	4.98 (SD = 2.27)
Adult trauma	7.99 (SD = 4.94)
Trauma-related diagnoses	
Burns and corrosions confined to the eye and internal organs	1 (0.02%)
Burns and corrosions of the external body surface, specified by the site	24 (0.41%)
Burns and corrosions of multiple and unspecified body regions	18 (0.31%)
Certain early complications of trauma	30 (0.51%)
Complications of surgical and medical care, not elsewhere classified	5 (0.09%)
Effects of foreign body entering through a natural orifice	3 (0.05%)
Injuries involving multiple body regions	42 (0.72%)
Injuries to the abdomen, lower back, lumbar spine, pelvis, and external genitals	378 (6.44%)
Injuries to the head	993 (16.91%)
Injuries to the neck	161 (2.74%)
Injury from shoulder to fingers	980 (16.68%)
Injuries to the hip and thigh	763 (12.99%)
Injury from knee to foot	532 (9.06%)
Injuries to the thorax	611 (10.4%)
Injury of unspecified body regions	650 (11.07%)
Other and unspecified effects of external causes	25 (0.43%)
Poisoning and toxicity	9 (0.15%)
Emergency department disposition	
AMA	62 (0.97%)
Home or home serv	1,035 (16.16%)
Observation	377 (5.89%)
Floor	3,321 (51.86%)
Transfer	77 (1.2%)
Stepdown	603 (9.42%)
Intensive care unit operating room	869 (13.57%)
Died	60 (0.94%)

Patient characteristics

Age

Data were compared for significant differences among variables. There was a significant difference between the MOI and ages of patients in the sample (F(16) = 136.69, p < 0.001). People aged 15-24 and 25-44 had the highest instances of MOIs related to MVAs as an occupant (n = 212, 29.44% and n = 622, 26.33%, respectively) (Table 2). There were consistencies in the instances of MOI for falls among the total sample, with patients between the ages of 15-24 and 25-44 not having “falls” as their highest incidents for MOI. When evaluating differences in shift arrival time and age, there was a significant difference (χ(10) = 270.998, p < 0.001); the overnight indicated younger patients arrived at the ED, while older patients tended to arrive during the day shift (Table 3).

**Table 2:** Trauma activation level and demographics, significance testing for differences (n = 6,404). *: Removed from inferential analyses due to low count. **: Data formatted as n, percentage (n, %) for categorical data, and mean, standard deviation (x̄, ±). n: sample size; SD: standard deviation; MD: medical doctor; ED: emergency department; LOS: length of stay; MOI: mechanism of injury; MVA: motor vehicle accident; NEC: not elsewhere classifiable; AMA: against medical advice

Variable	Trauma activation levels 1, 2**	Trauma activation consult, trauma aware but not activated, or not activated**	Significance
Age	44.08 (SD = 24.61)	57.34 (SD = 28.75)	p < 0.001
Trauma activation time and arrival time			p < 0.001
Trauma activation level before arrival	608 (74.4%)	209 (25.6%)	---
Trauma activation level after arrival	1,834 (44.6%)	2,275 (55.4%)	---
Race			p < 0.001
American Indian/Alaska Native	1 (50.0%)	1 (50.0%)	---
Asian	25 (37.3%)	42 (62.7%)	---
Black	445 (56%)	349 (44%)	---
Native Hawaiian/Other Pacific Islander	0 (0%)	3 (100%)	---
Other	650 (47.2%)	728 (52.8%)	---
White	2,821 (68%)	1,330 (32%)	---
Ethnicity			p < 0.001
Non-Hispanic/Latino	1,856 (36.1%)	3,282 (63.9%)	---
Hispanic/Latino	590 (47.5%)	653 (52.5%)	---
Trauma type			p < 0.001
Blunt	2,273 (37.1%)	3,857 (62.9%)	---
Burns	28 (36.4%)	49 (63.6%)	---
Penetrating	150 (78.5%)	41 (21.5%)	---
Arrived from			p < 0.001
Clinical/MD office	7 (16.7%)	35 (83.3%)	---
Home	775 (22.9%)	2612 (77.1%)	---
Jail*	1 (100%)	0 (0%)	---
Nursing home	21 (13.2%)	138 (86.8%)	---
Referring hospital	215 (74.1%)	75 (25.9%)	---
Scene	1391 (63.9%)	787 (36.1%)	---
Supervised living	13 (11.9%)	96 (88.1%)	---
Urgent care	28 (12.1%)	203 (87.9%)	---
Transport arrival type			p < 0.001
Ground ambulance	2,089 (45.8%)	2,473 (54.2%)	---
Police	8 (66.7%)	4 (33.3%)	---
Private/Public vehicle/Walk-in	354 (19.4%)	1,470 (80.6%)	---
ED arrival time/Shift			p < 0.001
Day	706 (28.8%)	1601 (40.55%)	---
Night	1243 (50.71%)	1800 (45.59%)	---
Overnight	502 (20.48%)	547 (13.86%)	---
ED LOS (hours)	5.72 (SD = 4.35)	8.14 (SD = 4.68)	p < 0.001
MOI categories			p < 0.001
Other	1,018 (67.7%)	485 (32.3%)	---
Cut/Pierce	137 (5.59%)	188 (4.76%)	---
Drowning	16 (0.65%)	2 (0.05%)	---
Natural/Environmental	9 (0.37%)	101 (2.56%)	---
Other specified, class	22 (0.9%)	148 (3.75%)	---
Other specified, NEC	12 (0.49%)	17 (0.43%)	---
Other transport	18 (0.73%)	26 (0.66%)	---
Overexertion	1 (0.04%)	10 (0.25%)	---
Pedacyclist-Other	43 (1.75%)	65 (1.65%)	---
Pedestrian-Other	14 (0.57%)	2 (0.05%)	---
Poison*	1 (0.04%)	1 (0.03%)	---
Burn	24 (0.98%)	50 (1.27%)	---
Firearm	76 (3.1%)	7 (0.18%)	---
Machinery	2 (0.08%)	52 (1.32%)	---
Medical care*	0 (0.00%)	1 (0.03%)	---
Struck by or against	110 (4.49%)	348 (8.81%)	---
Fall	824 (33.62%)	2,591 (65.63%)	---
MVA	1,129 (77.6%)	326 (22.4%)	---
MVA-Other	708 (28.89%)	275 (6.97%)	---
MVA-Motorcycle	142 (5.79%)	26 (0.66%)	---
MVA-Pedestrian	279 (11.38%)	25 (0.63%)	---
Emergency department disposition			p < 0.001
AMA	55 (2.24%)	7 (0.18%)	---
Home or home serv	904 (36.88%)	131 (3.32%)	---
Observation	150 (6.12%)	227 (5.75%)	---
Floor	592 (24.15%)	2724 (69%)	---
Transfer	26 (1.06%)	51 (1.29%)	---
Stepdown	177 (7.22%)	426 (10.79%)	---
Intensive care unit or operating room	490 (19.99%)	379 (9.6%)	---
Died	57 (2.33%)	3 (0.08%)	---
Death	57 (2.3%)	3 (0.1%)	χ(1) = 82.39, p < 0.001
Hospital complications	149 (34.2%)	287 (65.8%)	p = 0.36

**Table 3:** Age groups and demographics. ED: emergency department; MVA: motor vehicle accident

Variable	Age groups: 0–14 (n, %)	15–24 (n, %)	25–44 (n, %)	45–64 (n, %)	65–84 (n, %)	85+ (n, %)
Patient ED arrival time						
Day	220 (9.5%)	173 (7.5%)	383 (16.6%)	516 (22.3%)	629 (27.2%)	390 (16.9%)
Night	488 (16.0%)	367 (12.1%)	548 (18%)	645 (21.2%)	613 (20.1%)	383 (12.6%)
Overnight	58 (5.5%)	180 (17.2%)	294 (28%)	194 (18.5%)	187 (17.8%)	136 (13%)
Mechanism of injury						
Other	275 (18.30%)	299 (19.90%)	477 (31.70%)	347 (23.00%)	88 (05.80%)	20 (01.30%)
Cut/Pierce	17 (05.20%)	63 (19.30%)	132 (40.40%)	98 (30.00%)	15 (04.60%)	2 (00.60%)
Drowning	6 (33.30%)	5 (27.80%)	2 (11.10%)	2 (11.10%)	3 (16.70%)	0 (00.00%)
Burn	9 (12.20%)	8 (10.80%)	13 (17.60%)	17 (23.00%)	14 (18.90%)	13 (17.60%)
Firearm	4 (04.80%)	27 (32.50%)	44 (53.00%)	4 (04.80%)	4 (04.80%)	0 (00.00%)
Machinery	0 (00.00%)	4 (07.40%)	20 (37.00%)	23 (42.60%)	6 (11.10%)	1 (01.90%)
Natural/Environmental	7 (06.30%)	9 (08.10%)	30 (27.00%)	22 (19.80%)	22 (19.80%)	21 (18.90%)
Other land transport	7 (16.30%)	3 (07.00%)	7 (16.30%)	10 (23.30%)	10 (23.30%)	6 (14.00%)
Overexertion	1 (09.10%)	1 (09.10%)	2 (18.20%)	4 (36.40%)	2 (18.20%)	1 (09.10%)
Pedacyclist-Other	12 (11.10%)	6 (05.60%)	18 (16.70%)	18 (16.70%)	29 (26.90%)	25 (23.10%)
Pedestrian-Other	1 (07.70%)	2 (15.40%)	6 (46.20%)	2 (15.40%)	2 (15.40%)	0 (00.00%)
Struck by or against	57 (12.40%)	62 (13.50%)	103 (22.50%)	91 (19.90%)	87 (19.00%)	58 (12.70%)
Fall	348 (10.20%)	102 (03.00%)	258 (07.60%)	663 (19.40%)	1,183 (34.60%)	863 (25.30%)
MVA	137 (09.40%)	318 (21.90%)	480 (33.00%)	339 (23.30%)	155 (10.70%)	26 (01.80%)
MVA-Other	136 (13.80%)	124 (12.60%)	191 (19.40%)	204 (20.80%)	206 (21.00%)	122 (12.40%)
MVA-Motorcycle	21 (12.50%)	17 (10.10%)	32 (19.00%)	40 (23.80%)	34 (20.20%)	24 (14.30%)
MVA-Pedestrian	48 (15.80%)	43 (14.10%)	56 (18.40%)	56 (18.40%)	59 (19.40%)	42 (13.80%)
Mortality (“Yes”)	0 (00.00%)	8 (13.30%)	21 (35.00%)	15 (25.00%)	9 (15.00%)	7 (11.70%)
Hospital complications	2 (0.5%)	14 (3.2%)	42 (9.6%)	106 (24.3%)	156 (35.8%)	116 (26.6%)

Activation Levels

The sample’s activation levels were tested for ordinal and continuous levels, as well as into a dichotomous variable to test for differences among groups of higher levels of activation and lower levels of activation or trauma response. When testing for differences in patients’ age, there was a difference in activation level both with the groups as categorical levels (N, C, 2, 1) (F(3) = 298.76, p < 0.001), as well as when the data were treated as dichotomous (high trauma alert and low trauma alert) (t(5785.45) = 18.58, p < 0.001). Additionally, there was a difference in patients’ ED length of stay and their activation level (t(5473.06) = 26.86, p < 0.001). Differences were also noted among the ED arrival times for shifts and activation levels (χ(18) = 1582.89, p < 0.001), with a higher difference in activation levels during the day shift. Patients with higher activation levels were typically younger (44.08 average years of age; SD = ±24.61) compared to older patients (57.34 average years of age; SD = ±28.75) who had low levels of trauma alerts (t(5785.45) = 18.58, p < 0.001). Trauma level activation (higher vs. lower) was significantly different among MOI categories (χ(16) = 1585.23, p < 0.001). Patients who had a fall as their MOI were in lower activation levels when testing activations both as ordinal (t(5373.16) = 16.73, p < 0.001) and dichotomous data (χ(1) = 623.17, p < 0.001).

Mortality and Complications

There was a statistically significant difference between age and mortality; patients who were between the ages of 25 and 44 were more likely to have an ED disposition of “death” (n = 21, 35.00%) (χ(5) = 17.603, p < 0.05). When observing hospital complications within the different age groups, patients between the ages of 65 and 84 years had higher instances of complications (n = 156, 35.8%) (χ(5) = 191.794, p < 0.001). Additionally, patients with an MOI of “MVA-Other” had higher instances of mortality (n = 19, 32.20%), which was significantly different when compared with other MOIs (χ(16) = 172.291, p < 0.001). Patients, however, were more likely to encounter a hospital complication when they were at a lower activation level (n = 287, 65.8%), which was significantly different compared to patients who did not (χ(1) = 3.384, p = 0.36).

Geriatric Patients With Falls as Their Mechanism of Injury

There were 2,046 patients accounting for 31.9% of the sample who were aged 65+ years with a fall as their MOI. Between patients who were 65+ years with falls and those who were not, there was a significant difference in activation level (t(5859.472) =11.814, p < 0.001). When treated as continuous data, the trauma activation level mean for patients who were 65+ years with falls was 1.07 (SD = ±0.014) compared to the rest of the sample’s average of 1.32 (SD = ±0.015). Additionally, there was a significant difference in their ED length of stay (t(6,353) = -19.621, p < 0.001); patients who were 65+ years with falls as their MOI had a longer ED length of stay at an average of 9.303 hours (SD = ±0.104), while all other patients had an average ED length of stay of 6.846 hours (SD = ±0.070.) The groups were then compared when the data were treated as six groups based on geriatric and MOI and what their activation levels were when treated as a continuous variable (from highest average length of stay to lowest): <65 years with MVA (x = 1.91, SD = 0.677), 65+ years with MVA (x = 1.71, SD = 0.749), 65+ years with other MOI (x = 1.11, SD = 1.035), 65+ years with falls (x = 1.07, SD = 0.630), <65 years with a fall (x = 1.05, SD = 0.903), and <65 years with other MOI (x = 1.00, SD = 1.088). Significant differences were found when categorized into these six groups (F(5) = 234.494, p < 0.001). Patients who were aged 65+ years with a fall had significantly higher hospital complications when compared to all other patient groups (age by MOI) (n = 256, 58.70%) (χ(5) = 166.893, p < 0.001). When using the geriatric patient with falls grouping, the higher frequency of ED mortality occurred in the younger populations.

Subsample - ED Nurse Deployment for Trauma Patient Responses

**Figure 1:**
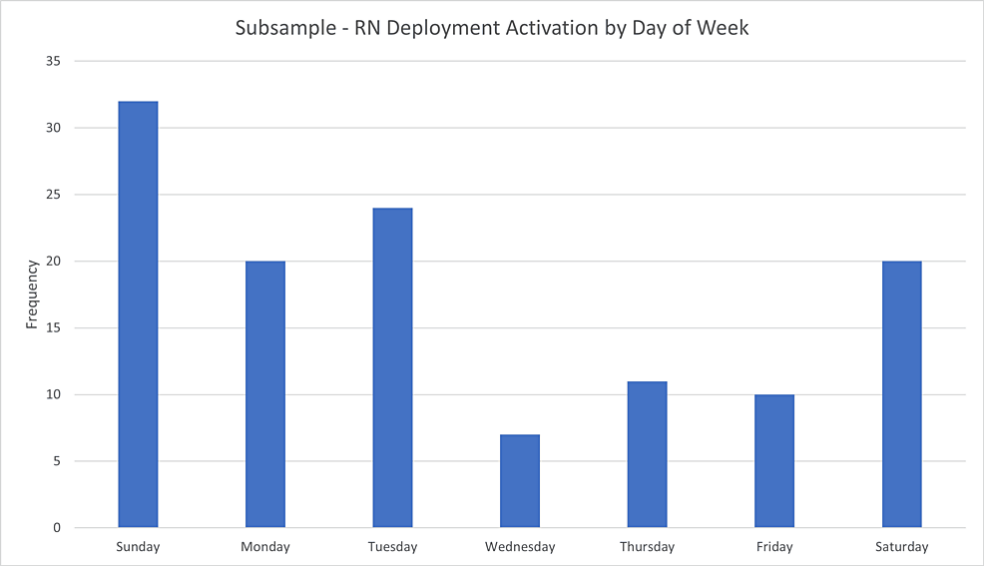
ED RN deployments by days of the week. ED: emergency department; RN: registered nurse

There were 124 instances where ED RNs were reallocated from their original ED non-trauma patients to respond to a trauma alert or activation for 37 patients with a trauma alert or response. The majority of these ED RN deployments were during the evening shift (n = 49, 39.5%). Trauma activations occurred most frequently on Sundays (25.8%) and least frequently on Wednesdays and Thursdays and Fridays (5.61% and 8.94%, respectively) (Figure 1). Trauma activations between Friday evening and Monday day shifts accounted for 51.3% of all trauma activations in the subsample. The trauma patients in the subsample were mostly adults (n = 29, 78.78%) with an average age of 40.56 years (SD = 30.84). There were nine (24.32%) pediatric patients. Of the MOIs for trauma patients in the subsample, 43.24% (n = 16, 45.95%) were falls. The majority of trauma patients were ESI level 2 (n = 28, 75.68%), and 26 (70.27%) patients were trauma activation level 2. Among the adult trauma patients ED RNs were deployed to in the subsample, the majority of MOI were falls (n = 15, 51.72%). The pediatric patients in the subsample who had ED RNs deployed to them had MOIs that were MVAs (n = 4, 44.44%), followed by others (n = 3, 33.33%), and falls (n = 2, 22.22%).  

ED RNs in the subsample were deployed to patients with a trauma alert for an average of 32.48 minutes (SD = ±33.15). In the subsample, in addition to the trauma response, there was an average of 3.35 ED RNs reallocated to each trauma patient. Additionally, the ED RNs were temporarily reallocated from an average of 4.09 non-trauma patients to respond to trauma patients, despite over a third of the trauma patients in the subsample being discharged home from the ED. Within this subsample, the majority of trauma patients were either discharged home (n = 13, 35.14%) or admitted to the floor (n = 13, 35.14%). No increased adverse events were observed among non-trauma patients or trauma patients within the subsampling frame.

## Discussion

Results from the study were similar to findings from previous studies [5,8]. Younger patients made up the higher percentage of patients who had a higher trauma activation level, which supported the evidence that there are disparities in trauma responses for different patient populations [6,7]. Findings supported a significant difference in activation levels for patients who were admitted to the ED with an MOI of falls; the majority of patients who had a fall were in a lower trauma activation level (n = 2,591, 75.87%) than those who did not have a fall and were in lower activation levels (n = 1,337, 45.34%). Comparing the MOI with age also indicated that older patients (65+) with an MOI of falls were more likely to have a lower trauma activation level and a longer ED length of stay [1,6,7].

The subsample of patients was from a time-motion study conducted continuously over two weeks in March and April 2021. The time-motion study was conducted because although the ED RN staffing levels within our institution were consistent with national ED staffing models, anecdotal feedback from the ED RNs suggested that a reassessment of the staffing models was needed to account for trauma patient responses. Within the subsample, there was a high ratio of patients who were assigned an ED RN and were subsequently left in the care of other ED RN team members when their primary ED RN was reallocated to a trauma patient. This supported previous studies’ suggestions that ED staffing models may need to incorporate coverage for non-trauma patients during a trauma response alert [1,4,12]. When analyzing whether registered nurses were deployed to lower-level acuity patients in the subsample, the majority of patients were a level 2, which would indicate a need for a revision to the trauma team or the ED staffing model for an appropriate trauma response [3,5]. However, of the patients (n = 26) who had a trauma activation level 2, 11 (42.31%) were admitted to the floor, and 11 (42.31%) were discharged to home. Only one (3.85%) patient in the trauma activation level 2 subset was admitted to the intensive care unit. This finding may support that there may be an overactivation trauma level that triggers a trauma team response that requires ED RNs to be reallocated from their original patient assignments [2,4].

Study limitations included the retrospective design, missing data, and the small sample size for the subset of trauma patients during the two-week time in motion study. A larger study comparing varied ED staffing models would provide further insights into the effect of trauma team deployment on patient outcomes and ED staffing models.

## Conclusions

Deployment of trauma resources occurred more frequently for younger patients and those presenting with penetrating or vehicle-related MOIs. ED nurses were deployed more frequently during the evening shift, with the majority of ED nurse deployment events occurring between the Friday night and Monday day shifts. Older patients with falls as their MOI received a lower-level trauma activation and required ED nurse deployment to augment trauma team resources deployed to these patients. Though quality patient-nurse ratios and staffing levels were observed continuously across the sampling frame, ED nurses did transfer care of non-trauma patients to other ED RNs on a daily basis to augment the trauma team response. As found in current evidence related to ED deployment of RNs for trauma activation, there may be geriatric patients with falls who require monitoring and attention even during a situation where a trauma alert is activated. Although instances of mortality are related to MVAs, geriatric patients who enter a facility with a fall as their MOI are still at risk for adverse outcomes and mortality. To prevent ED nursing resources from being reallocated, it is important to consider having a dedicated coverage system built into trauma team deployment, whether through staffing considerations or quality assurance provisions.
